# Research on Virtual Human Motion Control Based on Computer-Assisted Multimedia Simulation

**DOI:** 10.1155/2022/1902207

**Published:** 2022-04-20

**Authors:** Xiangzi He, Xinqin Jin, Jun Zheng

**Affiliations:** Harbin Institute of Petroleum, Harbin Heilongjiang, Harbin 150000, China

## Abstract

In order to reproduce realistic human motion in a virtual environment, a study of virtual human motion control based on computer-aided ergonomics simulation is proposed. First, we establish the required human body, workpiece, and production line simulation environment, and on this basis, we use OWAS, BSHA, and other analysis tools to carry out ergonomic simulation of virtual human motion simulation research, seek solutions to man-machine problems, and provide a new, efficient, and quantitative evaluation method for production line planning. The simulation results show that during the operation of virtual human 1, the ratio of the actual load on the left and right hands to the ultimate load has reached up to 70%. Therefore, it is necessary to revise the production line plan, and through analysis, it is concluded that the reason for the bad posture is due to the low height of the storage point and the machine tool. We believe that with continuous development, ProcessSimulate and ProcessDesigner will play a greater role in enterprise digitization.

## 1. Introduction

Ergonomics is also known as human engineering (HumanEngineering) and human factors engineering (HumanFactors). It is the use of research methods in subjects such as anthropometry, physiology, psychology, biomechanics, and engineering, an emerging edge subject that comprehensively conducts research on human body structure, function, psychology, and mechanics [1]. Since the 1960s, the rapid development of science and technology and the application of computer technology have injected new vitality into the research and application of ergonomics [2]. In the past two decades, with the continuous development of computer technology and network technology, virtual reality technology has developed by leaps and bounds and it was a huge success. Virtual reality technology represents abstract and complex computer data space as intuitive and familiar things for users. The virtual environment in the past mainly involved scenery, architecture, etc., and rarely involved people. However, with the in-depth development of virtual reality technology, the scope of the society's needs for virtual reality is also increasing and there is a demand for virtual people [3].

Virtual human is the representation of geometric and behavioral characteristics of a human in a computer-generated space. It is a graphic entity that is completely represented by a computer and looks like a real person [4]. As an emerging interdisciplinary subject, virtual humans involve virtual human modeling, animation design, computer graphics, physiology, psychology, and many research fields such as biomechanics, mechanics, mathematics, dynamics, robotics, and artificial intelligence; the theoretical foundation requires highly complex algorithm, and the practical application is strong. Therefore, the research of virtual humans is a basic application subject with theoretical significance and practical value. As a human-computer representation, the fidelity and immersion of the virtual human in the virtual environment determine the effect achieved [5]. With the increasing advancement of virtual human technology, virtual people have been widely used in engineering, virtual meetings, interaction, monitoring, virtual environments, games, training, education, military training, product design, maintenance, etc. In this way, the study of virtual humans has gradually become one of the very important research fields in computer science. The research on the motion control of virtual humans is a very important content in the research of virtual humans, and it plays an extremely important role in realizing the immersion, realism, and interactivity of the simulation system. Chen et al. proposed to improve the human-machine cooperation of the lane departure assistance (LDA) system. A man-machine sharing control strategy based on hybrid system theory is proposed. By considering the discrete and continuous state and time-varying longitudinal speed of the vehicle, the mixing system is formalized as a hybrid automation and a shared control strategy is established to manage human-computer interaction. The powerful gain scheduling energy-peak method is used to design the auxiliary system controller. By solving the linear matrix inequality (LMI), the D-stability and guarantee of the system are also studied. Through Carim/Simulink's common mode and loop (HIL) experiment, we can evaluate the proposed human-machine shared control method. The results show that this method can effectively keep the vehicle in the lane and proves good human-machine coordination [6].

Based on this research, first we introduced the existing mainstream ergonomic analysis methods, then a brief introduction to the simulation analysis software ProcessSimulate and ProcessDesigner is provided, an actual production line of a company is taken as an example to illustrate the implementation process of ProcessSimulate and ProcessDesigner in ergonomic applications, and finally, the production line is modified according to the obtained analysis report to give the optimized results.

## 2. Research Methods

### 2.1. Virtual Human Motion Model Based on Kinematics

Kinesiology is the subject of describing human movement. It only discusses the details of the movement itself and does not involve the causes of movement (internal and external forces) [7]. The so-called kinematics-based model means that the motion of an object is independent of the force that generates the motion, and its parameters include the position, velocity, and acceleration of the object. For example, in the H-Anim standard, three types of nodes (Node) are used to represent a virtual human model: they are the center of gravity of the human body, the joints of the human body, and the bone segments of the human body and divide the whole person into 1 person's center of gravity, 77 joints, and 47 bone segments. In the kinematics-based model, the complete kinematic parameters of any human skeletal segment require a total of 15 parameters that change over time; these kinematic parameters mainly include the position of the body core of the human bone segment, the body core velocity of the human bone segment, the body core acceleration of the human bone segment, the angle of the human bone segment, the angular velocity of the human bone segment, the angular acceleration of human bone segment, etc. Therefore, the human body's motion parameters are very complicated. In order to reduce the complexity of the research, usually, when describing specific sports, they are reasonably simplified, and in this way, only a small part of many motion parameters can be used to complete the description of this motion [8]. At present, the motion models of virtual humans in many types of software are based on kinematics; in the process of using these types of software to realize the animation of virtual humans, it requires the user to interactively set some key parameters and interpolate these key parameters to generate and control the movement of the object; the change of these parameters only relates to the size of each parameter value at each moment and the speed of change.

### 2.2. Ergonomics Analysis Theory

At present, the number of ergonomic research theories is increasing. According to the analysis of statistical results, more than 60 ergonomic methods have been used, including static force analysis, low back force analysis, job posture analysis, field of view analysis, fatigue recovery analysis, comfort level analysis, NIOSH analysis, RULA posture analysis, and OWAS analysis. Two ergonomic analysis theories with more applications are introduced in the following.

#### 2.2.1. OWAS Analysis Theory Based on Human Operation Posture

OWAS (ovako working posture analyzing system) is used to distinguish the body posture of people at work, grade it according to the degree of musculoskeletal injury that may be caused by the posture, and provide a reference for researchers to improve the worksite [9]. In the daily workspace design analysis, OWAS is considered to be an effective and easy-to-implement method for man-machine analysis of countless operating postures in various work scenarios; therefore, it is widely used in the industrial field.

OWAS mainly analyzes the posture elements of the human body's back, arms, legs, head, and 1 negative important element, and then according to the coding method of the above 5 items, it is displayed according to a specific permutation and combination. By analyzing the interaction between the five elements, the fatigue level of the human body posture is evaluated. OWAS divides the urgency of the work posture to be improved and the fatigue level of the work posture into 1∼4. See Table 1 for its grade classification.

#### 2.2.2. BSHA Analysis Theory Based on the Single Hand Lift of the Human Body

BSHA (Burandt–Schultetushand–Arman analysis) obtains the maximum allowable load that the arm system can bear by analyzing the human body's one-handed snatch work, and compares it with the actual load to reach the conclusion whether the one-handed operation is safe. Among them, there are four main factors that affect the results of BSHA analysis, that is, human factors, work-related parameters, load-related parameters, and actual load size [10]:

1. Human factors include workers' gender, age, and training level, and they determine the coefficient *P*_1_ (male = 1.0, female or male and female = −0.65), *T*_lim_ (25∼65 years old, coefficient is 0.80∼0.65), and *P*_2_ (average level = 1.0, better = 1.2, good = 1.4).
2. Job-related parameters include job type (dynamic or static), 4 aspects of working frequency and time, and arm position. These factors determine the static work factor *T*_stat_ and the arm's own mass *F*_*a*_.
3. Load-related parameters include load direction, lifting height, forearm posture, the angle between the upper arm and the forearm, and the position of the hand relative to the body. There are 5 items, and together, they determine the theoretical value *F*_max_ of the maximum load.

Combined with the influencing factors given above, workers working with one hand can withstand the maximum load ratio of *F*_act_/*F*_per_. The static load that the hand-arm system can bear is shown as follows:

(1) $$\begin{array}{c} F_{\text{per}}=\left[P_{1}P_{2}\min\left(T_{\text{stat}},T_{\lim}\right)\left(F_{\max},F_{a}\right)\right]-F_{a}. \end{array}$$

The dynamic load that can be withstood is shown as follows:

(2) $$\begin{array}{c} F_{\text{per}}=P_{1}P_{2}\min\left(T_{\text{dyn}},T_{\lim}\right)F_{\max}. \end{array}$$

When the actual load of the operation is greater than *F*_per_, it indicates that this task will cause damage to the human body and corresponding improvement measures need to be taken. BSHA tools can effectively eliminate hidden dangers when working with one hand.

### 2.3. Examples of Ergonomics Analysis

We take an example to illustrate the realization process of ProcessSimulate and ProcessDesigner in ergonomic analysis [11]. Using a virtual person on a production line designed by a company as the object of analysis and research, we use ProcessDesigner to organize and manage the process resources of the production line and ProcessSimulate's Human module to model, simulate, and optimize it.

#### 2.3.1. System Specification

This production line does not have fully automated manufacturing capabilities, and many stations require manual operations. The station composition of the study is 5 operators and 5 stations. Virtual person 1 needs to take the workpiece from storage point 1 to the product from the previous production line to station 1 and install the workpiece into the fixture for processing; at the same time, the finished workpiece in station 1 needs to be unloaded from the fixture and transported to the buffer point for processing. The work content of virtual humans 2∼5 is similar to virtual human 1, so we will not go into details here. The loading, unloading, and processing time of each station is shown in Table 2.

### 2.4. Virtual Human Motion Control and Simulation

In the virtual workspace, the virtual person must be adjusted and controlled, such as working posture and exercise, to perform certain tasks. In fact, there are also many ergonomic 3D CAD software, such as Anybody, Sammie, Apolin, Boeman, Cyberman, CAAA, Com-biman, CrewChief, Deneb/Ergo, Ergoman, Ergo-Space, Jack, Tadaps, and safewor [12, 13]. Some of these software types provide very realistic three-dimensional human body models, but some only provide simple human contours. At present, in human-machine engineering, the commonly used methods of virtual human motion control and simulation mainly include the following.

#### 2.4.1. Direct Control

Direct control allows users to interactively control the position and orientation of geometric objects in the environment through input devices, for example, translation and rotation, so as to understand the operation result in real time. For the human body that is hinged together, the movement of the components will affect other parts; therefore, constraints are generally used to define the relationship between them, and constraint satisfaction is achieved through inverse kinematics algorithms.

#### 2.4.2. Key Frame Technology

Key frame technology is a simple and direct motion control technology, and it relies on the experience of the operator to manually determine the main picture (key frame); let the software use interpolation technology to insert the picture, that is, automatically generate the frames between the main poses. The generation of the intermediate frame is completed by the computer to complete the interpolation, and all the parameters that affect the screen image can become the parameters of the key frame, such as position, rotation angle, and texture parameters. Since the key frame interpolation does not consider the physical properties of the human body and the relationship between the parameters, the obtained movement does not necessarily meet the evaluation requirements [14, 15]. In ergonomic simulation, position interpolation and orientation interpolation are usually introduced. Position interpolation is realized by spline-driven interpolation and velocity curve interpolation. Spline-driven animation refers to first setting the object's movement trajectory or path and then specifying the object to move along the modified trajectory. By adjusting the shape and position of the path, the movement process of the human body can be changed [16].

#### 2.4.3. Kinematic Method

Kinematics includes forward kinematics and inverse kinematics. The positive kinematics method enables users to adjust the angle of joints in real time; the inverse kinematics method requires the user to specify the position and direction of the end joints, and the computer automatically calculates the angle of each intermediate joint [16–18]. Since the kinematics method only considers the kinematic characteristics of the object and does not consider the dynamic characteristics of the object and uses user interaction to determine some key frames to generate motion, this kind of movement cannot fully reflect the real movement of the object and it is difficult to realize the real ergonomics evaluation. Therefore, it is necessary to give dynamic characteristics to the moving object.

## 3. Result Analysis

### 3.1. Solution

**Figure 1:**
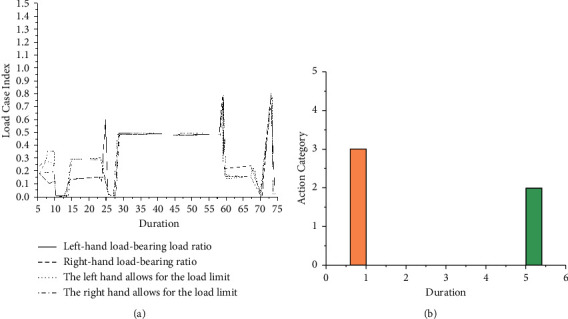
Virtual human 1 ergonomics analysis report. (a) BSHA report. (b) OWAS report.

It is planned to use ProcessDesigner in Siemens' Tecnomatix suite, and ProcessSimulate conducts research on the ergonomics of the production line. The Tecnomatix suite provides a digital manufacturing solution for SiemensPLMSoftware, by linking manufacturing planning including process layout planning and design, process simulation, and verification to manufacturing execution and product design, to realize the feasibility of using digital means to verify the manufacturing process of the product [19]. ProcessDesigner and ProcessSimulate were used to complete the research on the ergonomics of the production department. ProcessSimulate and ProcessDesigner are the components of the Tecnomatix suite. ProcessSimulate provides design, analysis, simulation, and optimization capabilities from factories to production lines and workstations. ProcessDesigner focuses on the management and simulation of resources and processes:

1. Build a model. The required 3D model format in Tecnomatix is a cojt or co file, and a 3D model can be established through Catia and other 3D modeling software first and then can be converted into a cojt or co file [20].
2. Build a resource library. The manufacturing system usually studied is a nonlinear discretization system, and it is necessary to establish product models, resource models (manufacturing equipment, raw materials, etc.) in ProcessDesigner, process model (process rules, manufacturing routes, etc.), and production management model (system restrictions and constraint relationships).
3. Build a product tree and product library. According to the BOM provided by the design department, enter ProcessDesigner according to a certain hierarchical structure and drag the parts in the product library into the corresponding product tree.
4. Before the simulation operation, all the digital and analog components must be placed in the ideal position, and the precise positioning of the digital and analog components can be completed by the relocate command in ProcessSimulate [21].
5. Human grabbing operations can be completed by “Auto Grasp” and “Grasp Wizard.”
6. Establishing a walking path can realize the self-motion of the virtual person. Complete the walking direction and walking posture through Path Editing.
7. ProcessSimulate provides a broad platform for the simulation of ergonomics; however, due to the complexity of the actual operation, many manual operation postures are quite complicated; therefore, when using ProcessSimulate, it is necessary to correct the posture of the human body many times to adapt to the actual situation. Complete the correction of human posture through HumanPostures > Joint Jog [17].
8. Complete the placement of objects through Place Object.
9. When designing the simulation operation, you can perform a trial run through the Operation toolbar, and after confirming that the simulation operation conforms to the actual production, save it in time and record the corresponding parameters for subsequent use.
10. After completing the simulation operation, the time of each operation unit needs to be adjusted. The Gantt chart can be used to intuitively understand the working tact time of each virtual person at each workstation [22].
11. Select the ergonomic analysis option, and set the parameters. Select Human > Ergonomics > Analysis Setup, check the OWAS and BSHA options, run the simulation program, and get the corresponding report [18]. Due to the similar work content of the virtual humans, only the ergonomic analysis of virtual human 1 is carried out separately, and the BSHA and OWAS reports are shown in Figure 1.

### 3.2. Result Analysis and Optimization

**Figure 2:**
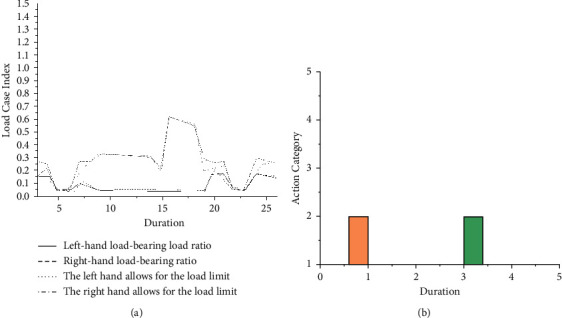
The ergonomic analysis report of virtual human 1 after the production line is improved. (a) Improved BSHA report. (b) Improved OWAS report.

From the above OWAS report, it is known that the posture level of virtual person 1 is 3 when picking up the workpiece, which explains that his body is endangered; according to the BSHA report, during the operation of virtual human 1, the ratio of the actual load on its left and right hands to its ultimate load has reached a maximum of 70%. Therefore, it is necessary to revise the production line plan. Through analysis, it is found that the reason for the bad posture is due to the low height of the storage point and the machine tool [23, 24]. Taking into account the actual production situation, the physical height of the storage point and station 1 can be increased by 50 cm and the OWAS report and BSHA report as shown in Figure 2 can be obtained.

From the above analysis report, we can know that the posture of virtual person 1 is greatly improved. Only the workstations and operations involved in virtual person 1 have been corrected accordingly. In practice, centralized analysis will be carried out based on the ergonomic analysis reports obtained by 5 virtual humans, and the production line planning will be evaluated and revised accordingly for each data [25].

## 4. Conclusion

The research and analysis of virtual human motion control based on ergonomics simulation was carried out around a certain engine machining production line, to guide production line planning. It can be seen from the actual case that dynamic virtual simulation based on process simulation and process designer can not only dynamically simulate the whole process of in-station operation but also perform various, ergonomic analyses of operating posture in real time. After discovering potential hazards, the simulation environment can be adjusted accurately and the ergonomics can be improved in a timely and accurate manner. The dynamic virtual simulation based on ProcessSimulate and ProcessDesigner represents the current international leading level in the field of ergonomics; it provides a brand-new evaluation method for enterprises to plan their production lines. On the basis of this paper, future research and improvement can be carried out in many aspects. Firstly, the existing virtual human model can be further improved. On the one hand, the bones and joints of the head, hands, and feet can be added as well as related constraints to further enrich the virtual human model. On the other hand, the muscle layer, skin layer, and clothing layer can be added on top of the bone layer to improve the visual effect and prepare for further man-machine evaluation.

## Figures and Tables

**Table 1:** OWAS fatigue classification.

Grade	1	2	3	4
Meaning	Acceptable	Minor injury	Significant damage	Serious injury

**Table 2:** Operating unit time of each station.

Station	Time		
	Loading time (s)	Cutting time (s)	Processing time (s)
	Feeding	Cutting	Processing
Station 1	9.6	8.3	28.9
Station 2	10.4	9.6	10.2
Station 3	5.6	5.3	34.7
Station 4	11.5	10.2	30.3
Station 5	5.6	3.3	13.2

## Data Availability

The data used to support the findings of this study are available from the corresponding author upon request.
